# Cross-clade neutralization potential of the plasma of antiretroviral naïve HIV-1 infected children from north India

**DOI:** 10.1186/1471-2334-12-S1-P87

**Published:** 2012-05-04

**Authors:** Prakash Somi Sankaran, Raiees Andrabi, Rajesh Kumar, Rakesh Lodha, Sushil K Kabra, Madhu Vajpayee, Kalpana Luthra

**Affiliations:** 1 Department of Biochemistry, All India Institute of Medical Sciences, New Delhi, Delhi, 110029, India

## Background

In this cross-sectional study, we evaluated the efficiency of the plasma of HIV-1 infected children from north India against a standard panel of pseudoviruses.

## Methods

We recruited 38 antiretroviral naïve HIV-1 infected children after getting written informed consent from their parents/guardians. The study was approved by the institute ethics committee. Neutralization efficiency of the patients’ plasma was tested against tier 2 pseudoviruses (3 clade C - ZM53, Du172.17 and Du156.12 and 3 clade B - RHPA4259.7, TRO.11 and SC422661.8) obtained from NIH AIDS Research and Reference Reagent Program by TZM-bl assay. The inhibitory dilution at 50% neutralization (ID50 titers) was determined by non-linear regression by the method of least squares. Correlation tests were carried out using Spearman rank correlation test.

## Results

The median age of the children was six years (range 1.5-14). The median viral load was 24000 RNA copies/ml (range <47-585000) and CD4 count was 655 cells/µl (range 131-2458). Cross neutralization was observed in 28.9% (11/38) of the children. Clade specific neutralization was observed in 47.4% (18/38) against clade C and 7.9% (3/38) against clade B while 15.8% (6/38) of the children did not show neutralization against any of the viruses. There was a significant positive correlation between viremia and neutralization efficiency against two of the viruses studied (Du172 r=0.49; p=0.007 and RHPA r=0.47; p=0.01).

## Conclusion

This is the first report on the neutralization efficiency of the plasma of HIV-1 infected Indian children against tier 2 pseudoviruses. Cross-clade neutralizing antibodies were observed in 29% of them.

